# Abaloparatide-induced pseudoxanthoma elasticum

**DOI:** 10.1016/j.jdcr.2025.03.029

**Published:** 2025-04-11

**Authors:** Sarah A. Amjad, Thais Pincelli, Shon E. Meek, Olayemi Sokumbi, James Keeling, Elisha M. Singer

**Affiliations:** a Mayo Clinic School of Medicine, Scottsdale, Arizona; b Department of Dermatology, Mayo Clinic, Jacksonville, Florida; c Department of Endocrinology, Mayo Clinic, Jacksonville, Florida

**Keywords:** abaloparatide, ABCC6, calcium, elastorrhexis, osteoporosis, parathyroid-related peptide, pseudoxanthoma elasticum

## Introduction

Pseudoxanthoma elasticum (PXE) or Grönblad-Strandberg syndrome is a rare, hereditary disorder characterized by ectopic calcification of highly elastic tissues of the eye, skin, cardiovascular, and gastrointestinal systems.^1^^,^^2^ PXE results from homologous or compound heterozygous mutations in the ABCC6 gene,^3^ and rare cases of drug induced-PXE have been reported.^4^ Typically, PXE is diagnosed in the second or third decade of life due to early skin findings.^1^ Herein, we present a case of PXE in a 56-year-old female six weeks after starting abaloparatide, a parathyroid related peptide receptor agonist, for treatment of osteoporosis.

## Case report

**Fig 1:**
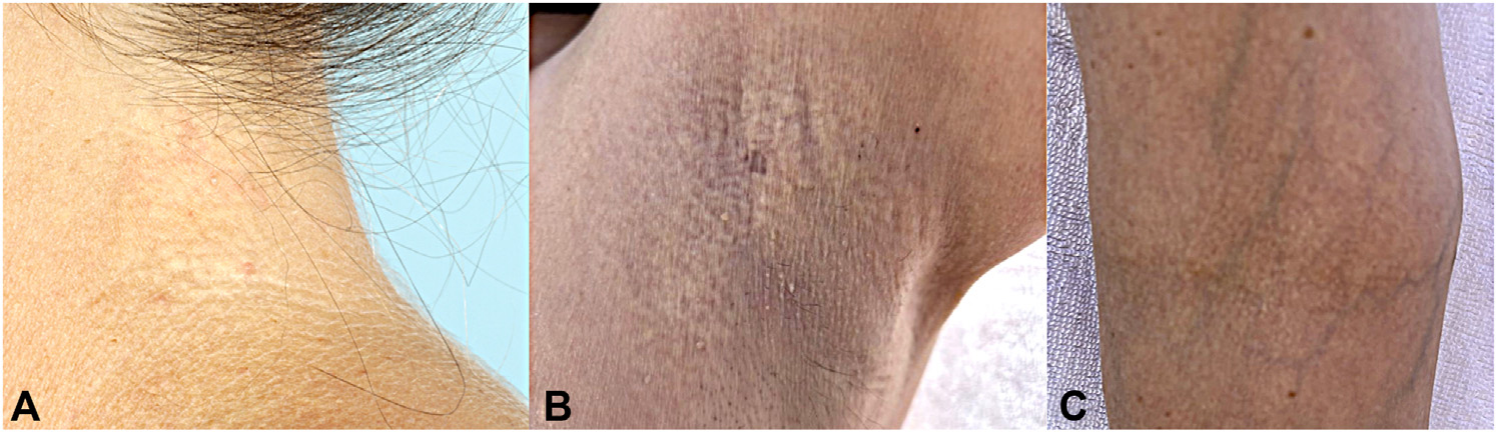
**A,** Photograph of the skin of the patient’s left neck. **B,** Photograph of the skin of the patient’s left axillae. **C,** Photograph of the patient’s right antecubital fossa. All three photographs demonstrate *yellow* papules coalescing into cobblestoned plaques.

**Fig 2:**
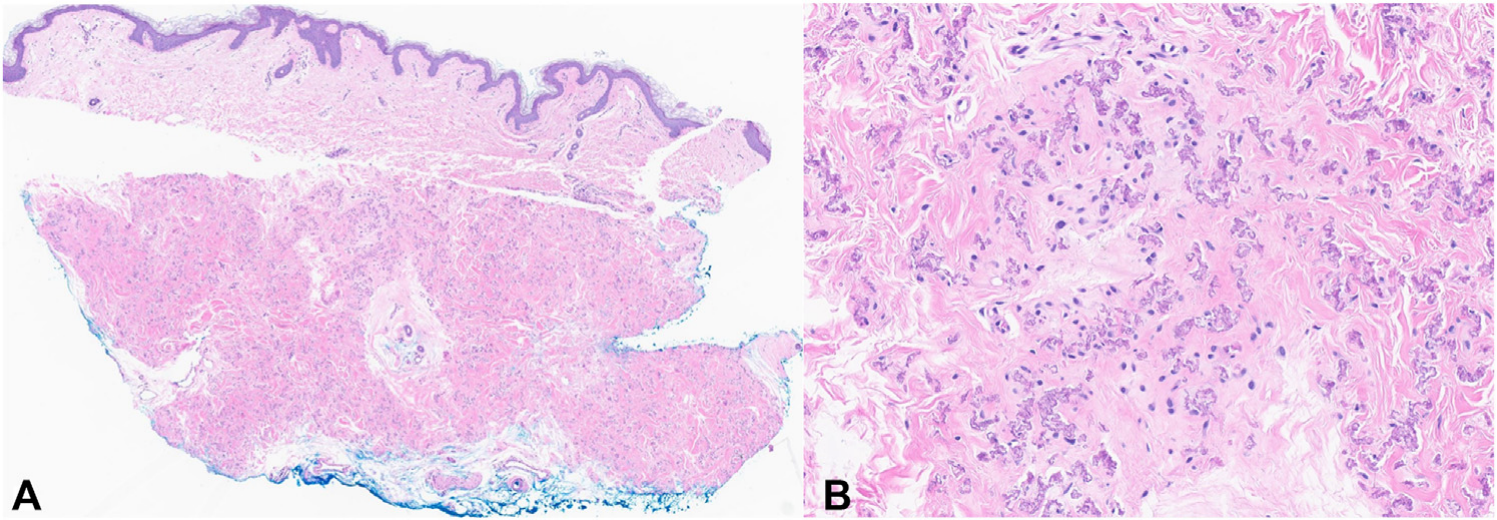
**A,** Punch biopsy obtained from the left anterior axillary crease demonstrates short and fragmented elastic fibers in the reticular dermis. **B,** The abnormal basophilic elastic fibers are readily identified (**A,** hematoxylin-eosin, original magnification ×4; **B,** hematoxylin-eosin, original magnification ×25).

A 56-year-old female with history of osteoporosis presented to dermatology for new-onset skin changes. She had been initiated on abaloparatide for osteoporosis, and 6 weeks later developed skin changes on her neck, axilla, and antecubital fossa (Fig 1). Prior to this, there were no skin abnormalities. Dietary history revealed no restrictions or supplementations, aside from daily vitamin D3 125 mcg. She took fexofenadine and diphenhydramine as needed. Laboratory data preceding abaloparatide therapy included normal serum creatinine (0.6 mg/dL), calcium (8.8 mg/dL), albumin (4.2 g/dL), 25 hydroxyvitamin D (44.0 ng/mL), parathyroid hormone (34 pg/mL), 24-hour urinary calcium (109 mg; 2.8 L), and negative celiac disease screening. Serum calcium and albumin measured one week into abaloparatide therapy remained normal (9.3 mg/dL and 4.6 g/dL, respectively). Histopathology of left anterior axillary crease biopsy demonstrated calcified and disorganized elastic fibers in the dermis, diagnostic of PXE (Fig 2). Genetic testing revealed one likely pathogenic variant mutation in the ABCC6 (Gain; Exons 11-19) and one variant of undetermined significance (c.1214C > T; p.Ala405Val). There was no family history of PXE. Although she denied visual changes, ophthalmology evaluation revealed angioid streaks, findings absent on retinal examination one week prior to starting abaloparatide. Cardiology noted no concerning cardiovascular findings. Given the temporal relation between abaloparatide initiation and the new onset of clinical findings and PXE diagnosis, the patient discontinued this medicine. Skin findings remain present, and she continues to follow with ophthalmology and cardiology.

## Discussion

PXE is a heritable elastic tissue disorder characterized by dystrophic calcification of elastic tissues. It is caused by a homologous or compound heterozygous mutation in the ABCC6 gene, which encodes adenosine triphosphate-binding cassette C6, a cellular transport protein.^3^ This protein is important to maintain serum plasma levels of inorganic pyrophosphate, an important anti-mineralization factor.^5^ Patients with PXE have 30% of normal plasma inorganic pyrophosphate levels, which leads to increased calcium phosphate supersaturation and ectopic deposition of calcium in elastic fibers of small and medium sized arteries in the eyes, skin, cardiovascular, and gastrointestinal systems.^3^^,^^5^ Patients present with yellow papules coalescing into plaques on skin flexural sites, resembling plucked chicken skin.^1^^,^^2^ Angioid streaks develop secondary to rupture of Bruch’s membrane which can lead to central visual loss. Peripheral artery disease complications can include hypertension, stroke, atherosclerosis, intestinal angina, and hemorrhage.^2^ Due to the skin findings, PXE typically is diagnosed in childhood or the second or third decades of life.^1^ The differential diagnosis of PXE includes pseudoxanthoma elasticum-like papillary dermal elastolysis, which presents in postmenopausal women with similar cutaneous findings, but with no evidence of dermal calcified and fragmented elastic fibers on histology, and without systemic involvement.^6^

Studies in animal models indicate that calcium and vitamin D supplementation accelerate disease progression in PXE.^7^ ABCC6−/− mice given calcium and vitamin D supplementation develop increased intravascular calcifications.^7^ PTH receptor agonists, including abaloparatide or teriparatide, directly increase the activity of osteoblasts more than osteoclasts when given as a daily subcutaneous injection to promote bone formation.^8^ Teriparatide, and to a lesser extent, abaloparatide, increase conversion of 25 hydroxyvitamin D to its active metabolite 1,25-dihydroxyvitamin D-3, increasing calcium absorption from the gastrointestinal tract.^8^^,^^9^ Abaloparatide and teriparatide both increase renal tubule reabsorption of calcium. Therefore, these drugs may lead to hypercalcemia in certain individuals.^9^ Although our patient’s calcium remained normal one week into therapy, abaloparatide can cause transient increases in serum calcium after subcutaneous injection, while still maintaining normal laboratory serum calcium levels.^10^ In our genetically predisposed patient, abaloparatide likely precipitated PXE despite not causing sustained hypercalcemia.

Our patient has a heterozygous mutation in the ABCC6 gene, which makes her a carrier of PXE.^3^^,^^5^ Although she has a variant of undetermined significance, this variant has not been reported in individuals with ABCC6-related conditions. Heterozygous carriers may be at increased risk for accelerated atherosclerosis and may display characteristic, comet-like retinal lesions, but no dermatologic disease has been reported.^3^ Furthermore, our patient had no skin findings consistent with PXE until she started abaloparatide, and her baseline ophthalmologic exam prior to abaloparatide therapy was without abnormal findings. Additionally, she did not consume a high calcium diet and was not on any estrogen, diuretics, or other osteoporosis medications prior to and during abaloparatide therapy. It is therefore highly likely that in the presence of a heterozygous ABCC6 mutation, abaloparatide induced sudden onset of PXE, through its impact on serum calcium homeostasis.

These findings are significant as they highlight a potential iatrogenic cause of PXE in a genetically predisposed patient, expanding the understanding of its etiology. Patients with D-penicillamine-drug-induced PXE present with similar cutaneous findings but without systemic involvement, and elastic fiber crosslinking is impaired due to penicillamine instead of dystrophic calcification.^4^ Although penicillamine discontinuation may lead to clinical resolution, some lesions may persist for several years.^4^ Our patient’s angioid streaks were present at her four-month follow-up ophthalmology evaluation, and her cutaneous findings persist, though long-term examinations are required to assess her prognosis.

Taken together, we recommend obtaining a thorough drug history in patients with new-onset PXE. We also suggest caution in prescribing parathyroid-related peptide analog medications in patients with a personal or family history of PXE. Additional research is necessary to further elucidate the precise mechanisms by which parathyroid-related peptide agonists induce PXE, and development of potential preventive strategies or alternative therapies that mitigate this risk is warranted. Understanding the interplay between pharmacological agents and genetic factors in the pathogenesis of PXE will be essential in developing comprehensive management approaches for affected patients.

## Conflicts of interest

None disclosed.

## References

[1] Li Q., Jiang Q., Pfendner E., Váradi A., Uitto J. (2009). Pseudoxanthoma elasticum: clinical phenotypes, molecular genetics and putative pathomechanisms. Exp Dermatol.

[2] Pfau K., Lengyel I., Ossewaarde-van Norel J. (2024). Pseudoxanthoma elasticum - genetics, pathophysiology, and clinical presentation. Prog Retin Eye Res.

[3] Nollet L., Campens L., De Zaeytijd J. (2022). Clinical and subclinical findings in heterozygous ABCC6 carriers: results from a Belgian cohort and clinical practice guidelines. J Med Genet.

[4] Ishak R., Abbas O. (2013). Penicillamine revisited: historic overview and review of the clinical uses and cutaneous adverse effects. Am J Clin Dermatol.

[5] Bartstra J.W., Risseeuw S., de Jong P.A. (2021). Genotype-phenotype correlation in pseudoxanthoma elasticum. Atherosclerosis.

[6] Atzori L., Ferreli C., Pilloni L., Rongioletti F. (2021). Pseudoxanthoma elasticum-like papillary dermal elastolysis: a mimicker of genetic pseudoxanthoma elasticum. Clin Dermatol.

[7] Bouderlique E., Tang E., Zaworski J. (2022). Vitamin D and calcium supplementation accelerate vascular calcification in a model of pseudoxanthoma elasticum. Int J Mol Sci.

[8] Yang Y., Tseng W.J., Wang B. (2023). Abaloparatide maintains normal rat blood calcium level in part via 1,25-dihydroxyvitamin D/osteocalcin signaling pathway. Endocrinology.

[9] Miller P.D., Hattersley G., Riis B.J. (2016). Effect of abaloparatide vs placebo on new vertebral fractures in postmenopausal women with osteoporosis: a randomized clinical trial. JAMA.

[10] Camacho P.M., Petak S.M., Binkley N. (2020). American Association of Clinical Endocrinologists/American College of Endocrinology Clinical practice guidelines for the diagnosis and treatment of postmenopausal OSTEOPOROSIS-2020 update. Endocr Pract.

